# The episodic evolution of fibritin: traces of ancient global environmental alterations may remain in the genomes of T4-like phages

**DOI:** 10.1002/ece3.730

**Published:** 2013-09-01

**Authors:** A V Letarov, H M Krisch

**Affiliations:** 1 Winogradsky Institute of Microbiology Russian Academy of Science117312, pr. 60-letiya Oktyabrya, Moscow, Russia; 2 Laboratoire de Microbiologie et Génétique Moléculaires, Centre National de la Recherche Scientifique UMR 5100 Université Paul Sabatier-Toulouse III118 Route de Narbonne Toulouse, 31062, Toulouse, Cedex 09, France; 3 Moscow Institute of Physics and Technology State University141700, Institutskiy lane 9, Dolgoprudny, Moscow Region, Russia

**Keywords:** Bacteriophage ecology, bacteriophage evolution, fibritin, modular evolution, T4-like bacteriophages, whiskers

## Abstract

The evolutionary adaptation of bacteriophages to their environment is achieved by alterations of their genomes involving a combination of both point mutations and lateral gene transfer. A phylogenetic analysis of a large set of collar fiber protein (fibritin) loci from diverse T4-like phages indicates that nearly all the modular swapping involving the C-terminal domain of this gene occurred in the distant past and has since ceased. In phage T4, this fibritin domain encodes the sequence that mediates both the attachment of the long tail fibers to the virion and also controls, in an environmentally sensitive way, the phage's ability to infect its host bacteria. Subsequent to its distant period of modular exchange, the evolution of fibritin has proceeded primarily by the slow vertical divergence mechanism. We suggest that ancient and sudden changes in the environment forced the T4-like phages to alter fibritin's mode of action or function. The genome's response to such episodes of rapid environmental change could presumably only be achieved quickly enough by employing the modular evolution mechanism. A phylogenetic analysis of the fibritin locus reveals the possible traces of such events within the T4 superfamily's genomes.

## Introduction

Phages, the extremely simple a-cellular parasites of the Eubacteria, are among the most numerous and diverse inhabitants of the biosphere (Clokie et al. 2011). In the classical view, the life cycle of virulent phages was defined by a rigidly encoded and inflexible developmental program; however, some feedback sensitive control mechanisms that can alter their developmental cycle were subsequently described (Krisch et al. 1974, 1977; Krisch and van Houwe 1976; Comeau et al. 2007; Golec et al. 2010; Moussa et al. 2012). Nonetheless, most phage adaptation to environmental pressures has occurred either by the accumulation of point mutations or by a lateral gene transfer of alien adaptive genes (Golais et al. 2013). Phages are particularly adept at co-opting sequences from diverse sources and exploiting them for their own needs (Arbiol et al. 2010; Murphy 2012). Phage lateral gene transfer can involve regulatory elements, individual genes, or large groups of genes of related function. Although the transferred sequences can remain largely unaltered, frequently they are gradually adapted to better accommodate the phage's specific needs. The requirements for such adaptive change were viewed by Darwin to be relatively constant in time. Hence, in his original formulation of the theory of evolution, adaptive change was envisaged as both a slow and relatively constant process. Subsequent discoveries, however, revealed that the evolutionary process was not as invariable as Darwin had believed and under exceptional circumstances the rates evolutionary change can be significantly accelerated. This realization resulted in a nontrivial revision of Darwin's theory that Eldredge and Gould (1972) called the “Punctuated Equilibrium Theory” which proposed that punctual events could force a transient but major increase in the rate of evolution. For example, the alteration of the global environment caused by the Cretaceous–Triassic impact event rapidly led to the extinction of the dinosaurs and their replacement by the mammals. This major event in the biosphere's history resulted in a substantial change in the rate of mammalian evolution. At least for simpler organisms such as phage, a switch from a mechanism of gradual mutational accumulation to a modular exchange mechanism involving lateral gene transfer offers a plausible mechanism by which such a rate shift could be accomplished.

The relative impact on phage evolution of gene transfer versus that of vertical gene divergence is known to vary significantly among different types of phage (Brussow and Kutter 2005). However, in all phages where extensive phylogenetic analysis has been done, both mechanisms appear to contribute (Brussow and Kutter 2005). Modular swapping in phage genomes is readily revealed by differences in phylogenies of the genes flanking the presumed module and the genes located within it. In opposition, a good correlation of the topologies of the phylogenetic tree of a series of neighboring genes suggests that they have all evolved by a vertical divergence mechanism (Filée et al. 2006).

A phylogenetic analysis of both the structural components and the replication genes of a large collection of T4-like phages have led Krisch and his coworkers to conclude that the vast majority of these genes have evolved primarily by a vertical divergence mechanism (Monod et al. 1997; Desplat and Krich 2003; Filée et al. 2006; Comeau et al. 2007, 2010). But the T4-like genome has a bipartite structure; interspersed within its conserved core of replication and morphogenesis genes are a series of smaller hyperplastic regions (HPRs). These HPRs contain numerous modular elements which frequently encode nonstructural phage genes that adapt its core genome to function effectively in diverse environmental niches (Filée et al. 2005; Nolan et al. 2006; Comeau et al. 2007, 2010; Millard et al. 2009; Sullivan et al. 2010). The diversity of the palette of HRP's genes presumably reflects the phage's need to rapidly and easily accommodate changes in its environment and lifestyle (Comeau and Krisch 2005; Weitz et al. 2005). Such genes reflect not only to the phage's current ecological situation (see Letarov 2012) but also its past adaptations.

There are a several notable exceptions in this bipartite organization of the T4-like genomes. For example, within the large segment of the genome that contains most of the virion's structural components, there is a locus that encodes the collar fibers (synonyms: whiskers, gp*wac*, and fibritin). Our previous work (Letarov et al. 2005) had indicated that this locus was much more variable than nearly all the other structural components of the virus. The only other virion components that have a greater level of genetic plasticity are the long tail fiber genes and their associated adhesins (Tétart et al. 1998; Trojet et al. 2011) which are the primary determinants of the phage's host range.

Phage evolution is believed to be mostly driven by the modest selective pressures that drive the phage to adapt to a slowly changing environment. There is little in the literature that characterizes the evolutionary response to rapid and transient (“punctual”) environmental events. When such dramatic environmental alteration occurs, adaptation must be achieved rapidly and if successful, the modified locus quickly dominates in the population. Here, we examine a series of long past evolutionary events that have mediated the acquisition (or deletion) of modular blocks of sequence and compare these to the more frequent and regular process of divergence by slow genetic drift. To do this, we analyze the diversity of the fibritin locus in T4-like phages and suggest that its evolutionary history could be the consequence of past dramatic change(s) in the phage's lifestyle or environment.

## Methods

The fibritin genes sequences were extracted from GeneBank by search for “fibritin” as a key word and by BLASTP search (http://www.ncbi.nlm.nih.gov) for the sequences related to T4 gp*wac* in bacteriophage genomes. The outputs of both searches were inspected manually to generate nonredundant set of the sequences. C-terminal regions of the fibritin homologs were analyzed by multiple BLASTP searches and the groups of related sequences (fibritin types) were identified. In some cases, the relatedness was confirmed by HHpred search (http://toolkit.tuebingen.mpg.de/hhpred). The sequences of gp23 (the major capsid protein) of the T4-type bacteriophages used in this analysis were then extracted from the GenBank and aligned using ClustalV algorithm using Megaline program in the Lasergene software package (DNA Star, Madison, WI). The gaps were manually removed from the sequences, and the gap-free sequences were realigned and the phylogenetic tree was generated.

### The organization, function, and genetic diversity of the fibritins

**Figure 1:**
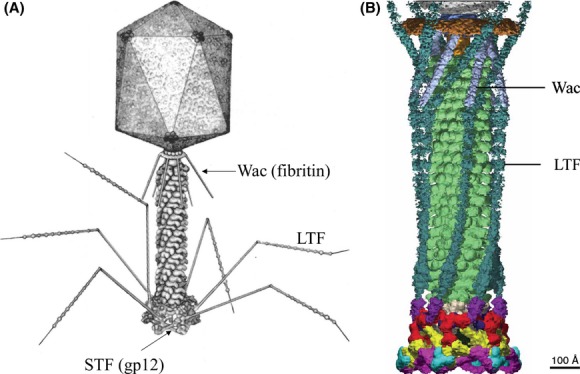
(A) Schematic representation of bacteriophage T4 particle. One of the six long tail fibers (LTF)s is shown interacting with the fibritin, all the other LTFs are in “down” position. (B) Cryo-EM base reconstruction of T4 tail. LTFs are in the “up” position. The interaction of the fibritin with the region of the LTF just below the knee is shown. Modified from Kostyuchenko et al. (2005) with permission.

**Figure 2:**
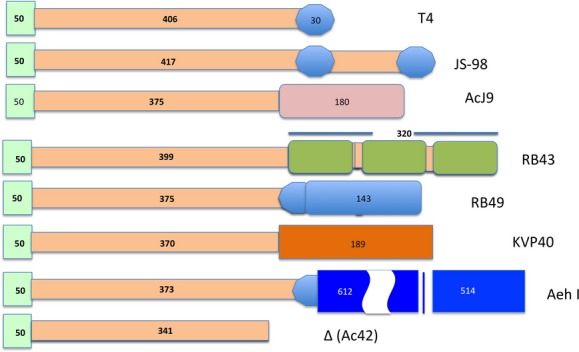
Schematic sequence representation of the main type of the fibritins. The numbers indicate the approximate lengths of the protein domains in aa. Only the sequences with the same color shading have significant homology to each other. For AehI-type fibritin, the presence of the additional nonhomologous gene encoding for a chaperone is indicated by a downstream blue box. Note that JS98 and T4 fibritin types may be considered as variants of a single fibritin type as one could be generated from the other by a single deletion or duplication of a segment of the gene. The spacer between two foldon-like motifs in JS 98-type fibritin may vary in length and its sequence has a detectable coiled-coil pattern. The GenBank accession numbers for the fibritin sequences of the phages indicated above are as follows: T4 – NP_049771.1; JS98 – AAU29287.1; Acj9 – YP_004010307.1; RB43 – AAP04365.1; RB49 – AAP04366.2; 44RR – NP_932503.1; Aeh1 – NP_944095.1; JS98 – YP_001595288.1; Ac42 – YP_004009533.1).

In bacteriophage, T4 fibritin is encoded by the gene *wac* (for *whiskers* *antigen* *control*) that forms the phage particle's collar structure consisting of six collar fiber whiskers (Fig. 1) (Follansbee et al. 1974; Kostyuchenko et al. 2005). In all the various T4-like phages where it has been examined, the fibritin homolog has been biochemically demonstrated to be a trimeric protein with a long fibrous shaft made up of segments of parallel triple-stranded α-helical coiled-coil structures that are frequently interrupted by small loops (Sobolev and Mesyanzhinov 1991; Efimov et al. 1994; Tao et al. 1997; Letarov et al. 2005). This shaft structure is flanked by a highly conserved 50 amino acids (aa) N-terminal domain that mediates the attachment of this fiber to the virion neck while in marked contrast, the C-terminal fibritin domain is extremely variable in size and sequence (Fig. 2). In T4, it is only a small 30 aa sequence that in addition to interacting with a motif located near segment long tail fibers (LTF)s knee also mediates the initiation of fiber's trimerization, and hence the orderly folding of the long fibrous central portion of the molecule (Efimov et al. 1994; Letarov et al. 1999). Interestingly, fibritin has a bifunctional role in the T4 life cycle. During phage morphogenesis it facilitates the attachment of the LTFs to the tail's baseplate structure (Fig. 1A). However, in the mature virion, the fibritin fibers act as part of the environmental sensing device by binding to each of the six LTFs and positioning them in the so-called “up” configuration that allows them to form additional protein–protein interactions with the tail shaft protein (Follansbee et al. 1974). In this tightly folded configuration (Fig. 1B), the LTFs are unable to initiate host adsorption (Follansbee et al. 1974; see also Letarov et al. 2005 and refs in this work). However, if environmental conditions become favorable for phage adsorption (Follansbee et al. 1974 and refs therein), the LTFs are released and absorption to an appropriate host can occur. Both morphological (Kostyuchenko et al. 2005) and genetic (Letarov et al. 2005) evidence indicate that the interaction between fibritin and the LTF fibers involves a small sequence within the C-terminal moiety of fibritin interacting with a motif located near the knee of the hinged LTF (Fig. 1B).

It remains unclear if all the known fibritin variants have the same bifunctional role as the T4 protein in both tail fiber attachment and environmental sensing; some could have different or additional functions. The sequencing of series of fibritin genes from phylogenetically distant subgroups of the T4 phages (T-even, PseudoT-even, and SchizoT-even [Letarov et al. 2005]) revealed that all the more distant fibritin sequences (those with less than 80% of overall aa identity) had alien sequences replacing their C-terminal domain (Efimov et al. 1994; Letarov et al. 2005; see also Latypov et al. 2007). However, both their N-terminal domains and major features of the coiled-coil shaft were well conserved among all these versions of the sequence. The recent availability of many more T4-like genomes sequences (Nolan et al. 2006; Comeau et al. 2007) has confirmed these observations and allowed the identification of additional variants of the fibritin C-terminal domain (Fig. 2). Such C-terminal sequences can be grouped into eight major alternative forms; nonetheless, even among these different forms there is some degree of divergence. For example, the JS98-like fibritins can differ in the length of the spacer between the two T4-like foldon motifs (Latypov et al. 2007), and the RB43-like fibritins may carry either a single Ig-like domain as in KP-15 (data not shown) or 3 Ig-like domains as in phage RB43 (Letarov et al. 2005). It has to be noted here that the JS98 and T4 fibritin types can be considered as a single variant, as T4 type could be generated from JS98 type by a deletion, similar to the above mentioned variants in the RB43-type phages.

Our biochemical analysis (Letarov et al. 2005; Latypov et al. 2007) of the T4, JS98, RB49, RB43, and AehI fibritins demonstrated that only the T4 and JS98 proteins are capable of folding themselves into trimers employing just the small T4-like foldon motifs. All the other fibritins require additional sequences either located within their expanded C-terminal domain or in a downstream gene for proper folding and trimerization (Letarov et al. 2005; Latypov et al. 2007). As an example, the phage AehI has two additional ORFs inserted immediately downstream of the fibritin sequence. These ORFs, annotated as ORFs Aeh1p218 and Aeh1p219 in GenBank, are found only in the fibritin locus of the AehI cluster of the sequenced T4-like phages (Letarov et al. 2005). The protein gp219 is insoluble when expressed from a chimeric plasmid and has no homolog in the other AehI-like phages, so it may be a pseudogene; however, gp218 homologs are present in the fibritin locus of all the AehI-related genomes that have been examined. The gp218 protein was easily overexpressed in *Escherichia coli* in a native, soluble form. Although the overexpression of AehI fibritin by itself resulted in the formation of insoluble inclusion bodies, in a double-expression system where both fibritin gene and gp218 were simultaneously produced, we obtained AehI fibritin that was in the soluble native conformation by the multiple criteria (a compact trypsin-resistant structure that formed SDS-resistant oligomers and had weak binding to hydroxylapatite columns (A. V. Letarov and H. M. Krisch, unpubl. data). Hence, we concluded that the AehI version of fibritin requires the downstream gp218 protein as a chaperone to correctly fold this unusually long molecule, which is twice the length of the T4 gp*wac*. The proper folding of the AehI fibritin was only obtained when the temperature of fibritin/chaperone expressing culture was 25°C or less (Letarov 2004); an observation that may explain why AehI phage growth is similarly temperature sensitive. The seven other variants that form the C-terminal domain can all apparently initiate fibritin's trimerization and correct folding without such a specific additional chaperone.

### The evolution of the fibritin

**Figure 3:**
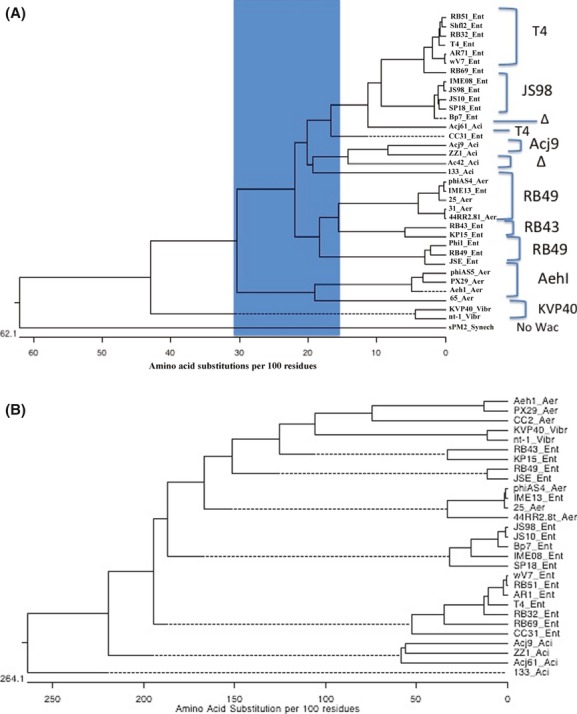
(A) The strong correlation between the fibritin types with the gp23 phylogeny. A phylogenetic tree of the major capsid protein (gp23) was constructed from an alignment generated by Clustal V algorithm. Dotted lines are inserted to correctly align these sequences. On the right of this gp23 phylogram, the phylogenetic clusters of the fibritins that correspond to the different C-terminal modules are indicated. Each of these types is closely associated with a single-branch gp23 tree. These fibritin clades are named arbitrarily after typical representative phage in the group. The Type △ grouping includes the phages with a deletion of the essential domains of the fibritin protein. Some phages closely related to those shown in the figure were not included in the figure to simplify the presentation, and their fibritins invariably belong to the same type as their relatives which are shown. The shaded box indicates the period when active modular swapping of the fibritin C-terminal domains occurred (for determination of the box's limits we consider JS98 and T4 types of the fibritin as variants of a single type for the reasons explained in the text). Note that four of five modular replacement events in the fibritin C-terminus occurred during the period marked by the box. Phage host ranges are indicated as follows: Ent, Enterobacteria; Aci, *Acinetobacter;* Aer, *Aeromonas;* Vibr, *Vibrio;* Synech, *Synechococcus*. (B) For comparison to the phylogenetic analysis in Panel 3a, this panel presents the tree of the conserved N-terminal structural part of the Fibritin gene that unlike the C-terminal module has evolved in a homogenous and regular fashion.

The comparison of diverse T4-like genomes (Fig. 3A) indicates that all the phages with the same type of the fibritin are otherwise quite closely related based on their gene 23 (major capsid protein) sequence which is a reliable and general phylogenetic marker gene for the T4 superfamily (Filée et al. 2006). The only exceptions are the phages with deletions in fibritin whose host range is essentially confined to *Acinetobacter* species, a host where the fibritin function may be nonessential. This tight coupling of the phylogenies of the numerous core genes of the genome with the fibritin C-terminal module was unexpected and rather puzzling. For example, the distribution of the C-terminal domains of both gp37 (distal subunit of the LTF) and gp38 (its associated adhesin) that are the major determinants of the phage host specificity have a more typical modular behavior in which they have been frequently and regularly subjected to modular shuffling events (Tétart et al. 1998; Trojet et al. 2011). As a result the LTF adhesin specifying domains have diverged much more facilely than the T4 core genome, not less, as the fibritin gene.

The evolutionary behavior of the fibritin C-terminal module is curiously atypical, although it experienced modular switching in multiple independent lineages during a period in the distant past (indicated by the blue shaded box in Fig. 3A); this ceased and the locus became effectively genetically fixed. We had previously assumed that the acquisition or the deletion of genes and protein domains by phages is essentially a rare but constant process, like evolutionary divergence *via* the slow and regular accumulation of point mutations. Fibritin's anomalous behavior could be explained by the occurrence of a period of very strong selection for a major alteration in the function of this locus. As several groups of the T4-like phages with different types of the fibritin all infect *E.coli* and its most closely related enterobacteria, it seems unlikely that modular switching within the fibritin locus involves just a change in the phage's host range. Rather, we suggest that it is more likely that the novel C-terminal fibritin domains encode functions that facilitate the phage's adaptation to alterations in its ecological niche. The nature of such a niche change remains unclear as we are currently unable to calibrate phage phylogeny to an absolute timescale. A dramatic change in the global environment could cause a considerable alteration in both the size and composition of the biosphere's bacterial population that would inevitably impact on the phage population by necessitating appropriate adaptions to the new set of conditions. Similarly, epic events like the possible invasion of the marine T4-like phages into a terrestrial ecosystem would require profound adaptive changes in the pioneer phage population. In a related, but less dramatic fashion, changes in the dominant groups of terrestrial vertebrates would probably also provoke significant alterations of the biosphere's enterobacterial population. The successful establishment of a T4-like phage population to a recently perturbed or a newly opened environmental niche must depend, at least in part, on the gene content of the nonconserved portion of its genome, including the fibritin gene. If the numerous versions of fibritin have dissimilar adaptive functions, the gene frequency of the most appropriate of fibritin will eventually predominate in the new niche and then undergo gradual evolution to improve its contribution to the phage's fitness. Thus, a subpopulation can emerge and become genetically separated from the rest of the T4-like superfamily. This newly emerged phylogenetic branch of the superfamily would be characterized by a highly selected group of adaptive genes, including the fibritin C-terminal module that it carries.

Some features of the newly characterized fibritins variants suggest that their function(s) may be different from those of T4 gene. For instance, the Ig-like domains that are present on RB43 fibritin (Letarov et al. 2005) are found in many phage proteins that are in direct contact with the external environment (Fraser et al. 2007). Such fibritins with Ig-like domains could actually have a role analogous to the adhesin functions associated with the T4 gp37 tail fibers. A fibritin encoded adhesin-like function might not directly mediate irreversible phage adsorption, but rather bring the virion and hence its LTF adhesin into sufficiently close proximity to the bacterial host's surface to initiate adsorption by the tail fibers adhesins. Such an accessory role for fibritin in adsorption could be particularly valuable in a situation where the bacteria density was exceptionally low such as immediately after a mass extinction event (Fraser et al. 2007). The existence of such “accessory” adhesins has recently been demonstrated for *Caulobacter crescentus* phages CbK and Cb13 that interact with the host flagellum via the proteins attached to their capsids and take the advantage of the flagellum rotation to locate the ultimate receptor target (Guerrero-Ferreira et al. 2011). Similarly, the involvement of the capsid decoration proteins in the adsorption process has been shown for *Salmonella* phage P22 (L. Gogokhia, pers. comm.). It is perhaps relevant that the organization of the AehI fibritin gene with its exceptionally long (more than 900 aa.) fibrous domain mimics the structure of many of phage tail fibers. Taken together with the weak homology of its large C-terminal domain to diverse phage tail fiber proteins, this argues in favor the involvement of this version of fibritin in the AehI phage's host recognition process (Letarov 2004; Letarov et al. 2005).

## Discussion

Given the equally convincing evidence for extensive modular shuffling of C-terminal domain of fibritin in the past and the absence of recent mobility, it seems that the selective pressure that once existed for swapping this domain has now vanished. The ubiquity and enormous population size of the T4 superfamily argue that whatever the transitory responsible event was, its impact on the biosphere must have been global. As an example, mass extinction events are among the obvious candidates, as there have been a series of at least five such major and more minor catastrophes during the last 700 Myr (Raup and Sepkoski 1982). Calibration of the T4 superfamily molecular clock might allow us to better assess if these or other more recent environmental events (e.g., ice ages, periods of global warming, etc.) caused the mobility of the fibritin C-terminal domain. Another interesting question for further investigation concerns whether additional adaptive genes were acquired by the diverse T4 phages at the same time and for the similar purposes as the novel fibritin domains.
